# Olfactory dysfunction and increased systemic immune inflammation index in patients with atrial fibrillation

**DOI:** 10.17305/bb.2026.13722

**Published:** 2026-02-17

**Authors:** Ercan Akşit, Ahmet Köder, Uğur Özkan, Cihan Öztürk

**Affiliations:** 1 Department of Cardiology, Canakkale Onsekiz Mart University Faculty of Medicine, Canakkale, Türkiye; 2 Department of Otorhinolaryngology, Trakya University Faculty of Medicine, Edirne, Türkiye; 3 Department of Cardiology, Trakya University Faculty of Medicine, Edirne, Türkiye

**Keywords:** Atrial fibrillation, olfaction, Sniffin’ Sticks, systemic immune inflammation index

## Abstract

Atrial fibrillation (AF) is a common arrhythmia with substantial morbidity and a need for accessible markers that reflect disease burden, while olfactory dysfunction and chronic inflammation may share overlapping vascular and neuroinflammatory pathways. We aimed to evaluate olfactory function in patients with AF and to explore the role of systemic inflammation using the systemic immune-inflammation index (SII). In this single-center case–control study, 85 consecutively enrolled adults (AF group, *n* ═ 43; control group, *n* ═ 42) underwent olfactory assessment with the Sniffin’ Sticks Extended Test, including odor threshold (OT), odor discrimination (OD), and odor identification (OI), from which the threshold–discrimination–identification (TDI) score was derived; SII was calculated from same-day complete blood counts. Compared with controls, patients with AF had higher SII (878 [368–5769] vs. 503 [243–1450], *P ═* 0.007) and lower OT (4 [1–8] vs. 5 [2–10], *P ═* 0.001), OD (7 [4–12] vs. 13 [8–16], *P <* 0.001), OI (7.7 ± 2.6 vs. 12.6 ± 1.7, *P <* 0.001), and TDI scores (19 [10–29] vs. 29.5 [25–39], *P <* 0.001). Within the AF group, olfactory performance was inversely associated with symptom severity assessed by the European Heart Rhythm Association (EHRA) classification, and TDI was negatively correlated with SII. These findings indicate that AF is associated with impaired olfactory function and elevated systemic inflammation, supporting olfaction and inflammatory indices as potential correlates of symptom burden that warrant confirmation in larger prospective cohorts with standardized rhythm monitoring.

## Introduction

Atrial fibrillation (AF) is one of the most prevalent cardiac rhythm disorders, associated with significant morbidity and mortality, and imposes a considerable economic burden on healthcare systems [1, 2]. Current data indicate that over 55 million individuals globally have AF, with approximately 365,000 fatalities attributed to the condition each year [3]. AF is associated with a fivefold increased risk of heart failure and stroke, as well as an approximately twofold increase in myocardial infarction [1]. The economic impact of AF on healthcare systems in Europe has been quantified at €66,242,359 [4]. Furthermore, the paroxysmal nature of AF and the fact that 8.6% of cases are asymptomatic underscore the urgent need for innovative diagnostic tools for this condition, which carries high mortality and morbidity rates [5]. The high recurrence rates following ablation further emphasize the necessity for advancements in both diagnosis and treatment [6–9]. Numerous studies have also demonstrated a strong association between AF and cognitive disorders, as well as neurodegenerative diseases [10–14].

The sense of smell is integral to daily life, and its impairment can profoundly affect an individual’s quality of life and overall health. Olfactory dysfunction may arise from various underlying conditions, including neurodegenerative diseases, viral infections, head trauma, and exposure to certain chemicals [15]. Additionally, olfactory dysfunction is frequently linked to underlying pathophysiological mechanisms such as systemic inflammation, vascular dysfunction, and neurodegenerative processes, all of which contribute to the pathogenesis of AF [16, 17]. Cranial magnetic resonance imaging (MRI) studies have revealed compromised white matter integrity and small vessel disease in patients with both AF and olfactory dysfunction [18, 19]. The systemic immune-inflammation index (SII), an indicator of chronic inflammation, has been shown to predict AF and is also impaired in neurodegenerative diseases [20, 21]. Beyond being a marker of chronic inflammation, SII serves as a significant clinical predictor of increased short-term mortality in patients with sepsis [22]. Although the link between cardiovascular diseases and olfactory dysfunction has begun to be explored in recent years, no direct studies have specifically examined its association with AF [23–25].

Given this context, the primary objective of this study was to evaluate olfactory function in patients with AF. Additionally, the study aimed to investigate the relationship between AF and olfactory function while assessing SII in this patient group to better understand the role of chronic inflammation in this process.

## Materials and methods

### Study population

This investigation was designed as a case-control study, conducted concurrently in the cardiology and otorhinolaryngology outpatient clinics. A total of 85 patients were consecutively enrolled, comprising 43 in the AF group and 42 in the control group. The study was conducted at a tertiary hospital between September 2023 and June 2024.

### AF group definition

included patients with paroxysmal AF who had been previously diagnosed or were diagnosed with AF at the time of admission to the cardiology department. Paroxysmal AF was defined as AF that resolved spontaneously or through intervention within seven days of onset, in accordance with established guidelines. Diagnosis was confirmed through documented electrocardiographic (ECG) evidence: (1) a 12-lead ECG showing AF, (2) AF lasting ≥ 30 s on an ambulatory ECG Holter, or (3) AF confirmed by a cardiologist in hospital records [2].

### Control group definition

The control group was comprised of patients attending the cardiology outpatient clinic for routine check-ups. Current ECGs were evaluated for this group. Silent AF is infrequent in individuals under 65 years of age; therefore, current guidelines recommend ECG Holter monitoring for individuals aged ≥ 65 years with additional risk factors or for all individuals over 75 [2]. Consequently, control group participants were specifically selected from those under 65 years of age without a history of palpitations. The control group was matched for sociodemographic characteristics with the AF group but exhibited normal sinus rhythm (NSR) and had no history of AF, atrial flutter, other arrhythmias, or related symptoms such as palpitations.

**Figure 1.**
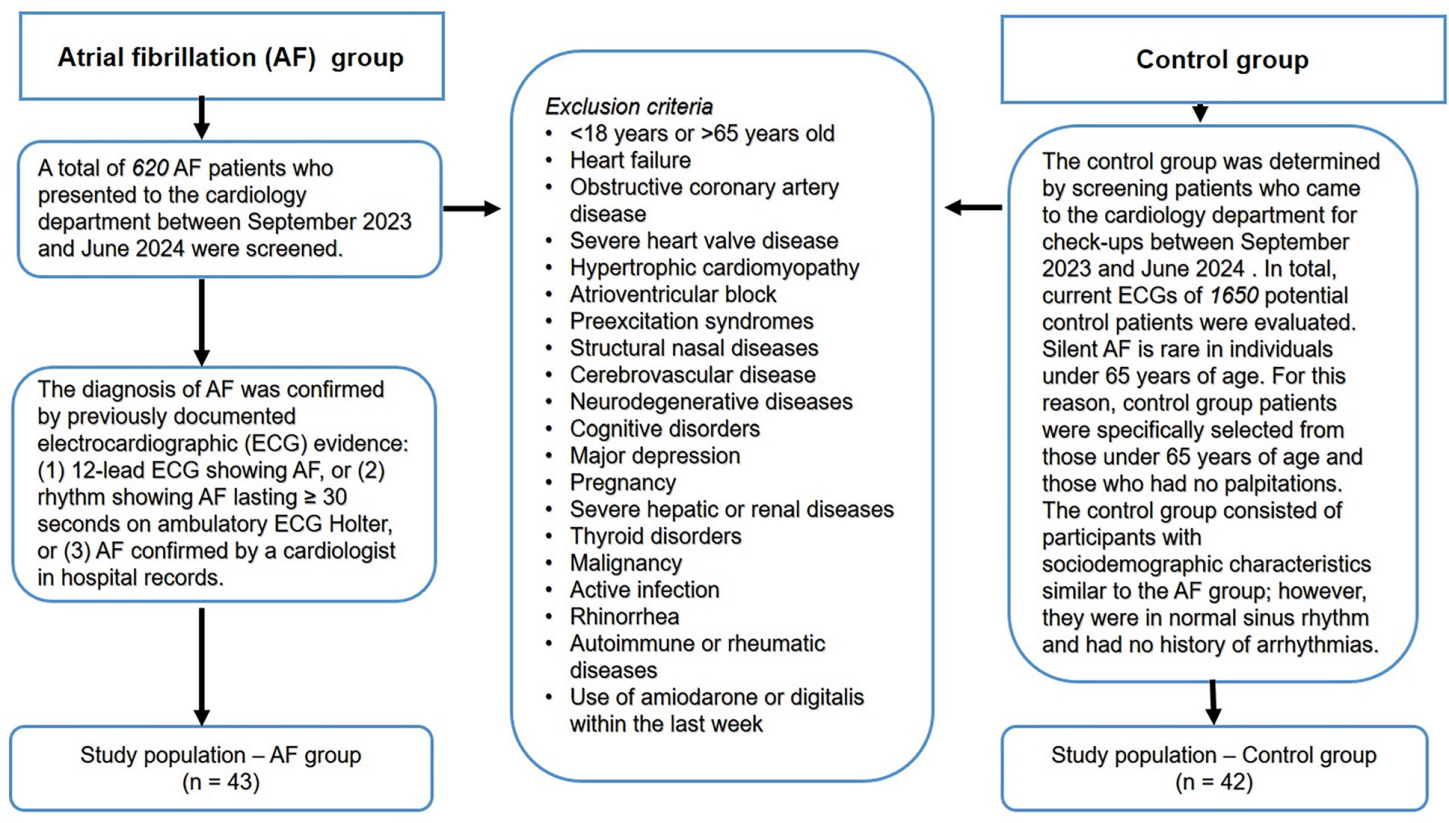
**Flowchart of the study design**.

Given that numerous conditions, particularly advanced age, affect olfactory function, patient selection was conducted meticulously, with detailed exclusion criteria established [26]. Patients younger than 18 years or older than 65 years, those with heart failure with reduced ejection fraction, obstructive coronary artery disease, severe heart valve disease, hypertrophic cardiomyopathy, a history of myopericarditis, and those with other arrhythmias—especially atrioventricular block and preexcitation syndromes—were excluded from the study [23–27]. Additional exclusion criteria included patients with structural nasal diseases (e.g., allergic rhinitis, septal deviation, choanal atresia, turbinate hypertrophy), cerebrovascular disease (ischemic or hemorrhagic), neurodegenerative diseases, cognitive disorders, major depression, pregnancy, severe hepatic and renal diseases, thyroid disorders, malignancy, active infections, rhinorrhea, and autoimmune and rheumatic diseases. Furthermore, patients who had used amiodarone or digitalis within the preceding week were also excluded, as these medications are commonly utilized in AF management and have been associated with reversible olfactory dysfunction [28, 29]. Although hypertension and diabetes mellitus can contribute to olfactory dysfunction, they are frequently present in the etiology of AF; thus, a comparable number of patients with these conditions were included in both groups. The flow chart of the study population is presented in Figure 1.

Clinical characteristics, demographic information, laboratory parameters, and physical examination findings for both groups were retrieved from the hospital’s automated system. Echocardiographic parameters were also acquired from patient files. Prior to olfactory testing, the ECG status revealed that 51 patients had sinus rhythm and 34 had AF rhythm. Since the study included patients with paroxysmal AF, those exhibiting sinus rhythm prior to the olfactory test were still classified in the case group. Based on initial electrocardiograms, patients were identified as having either AF or sinus rhythm. Left ventricular end-diastolic diameters, left ventricular ejection fractions, interventricular septum thickness, and estimated systolic pulmonary artery pressures were recorded from transthoracic echocardiography. Arterial blood pressure, pulse rates, weight, and height were documented for patients in both the AF and control groups. Body mass index (BMI) was calculated by dividing body weight (in kilograms) by the square of height (in meters) [30]. The symptom severity of AF patients was assessed using the European Heart Rhythm Association (EHRA) classification, which categorizes symptoms as follows: EHRA 1: no complaints, EHRA 2: mild symptoms that do not affect daily activities, EHRA 3: severe symptoms impacting daily activities, EHRA 4: disabling symptoms preventing normal daily activities [31].

### Assessment of olfactory function

Olfactory function was assessed using the Sniffin’ Sticks Extended Test (Heinrich Burghart, GmbH, Wedel, Germany). This test comprises three subtests that measure odor threshold (OT), odor discrimination (OD), and odor identification (OI) to evaluate olfactory function. Given that this study is the first to directly assess olfactory function in AF patients, the more comprehensive Sniffin’ Sticks Extended Test was utilized rather than the 12-item version [32, 33]. The odor tests were administered by a single otorhinolaryngology specialist, who was blinded to the study groups. All assessments took place in a controlled environment, with the testing conducted in a well-ventilated room at a temperature of 22 ± 2 ^∘^C and a relative humidity of 40%–60%, free from distracting environmental odors (e.g., perfume, disinfectant, cigarette smoke, food odors). Participants were instructed not to smoke or eat for at least one hour prior to testing, and none had active infections or rhinorrhea. All participants were asked to smell predefined odors using felt-tip pens (commonly known as *Sniffin’ Sticks*) measuring approximately 1.3 cm in diameter and 14 cm in length, each containing about 4 ml of scent-diffusing liquid. The scent pen was held about 2 cm from the participants’ noses, alternating between the left and right nostrils for three seconds before being withdrawn. The average olfactory test scores from both nostrils were taken for each OT, OD, and OI value. The threshold value was defined as the lowest concentration detectable from the odor sticks (maximum score: 16), while the discrimination value was determined by the number of correctly distinguished sets (maximum score: 16). The identification value was calculated based on the number of accurately identified scent sticks (maximum score: 16). The combined score from the three subtests yields the threshold-discrimination-identification (TDI) score (maximum score: 48). As previously established, TDI scores below 16 were classified as anosmic, scores between 16 and 30 as hyposmic, and scores above 30 as normosmic [34].

### Calculation of SII

Hemogram parameters were assessed on the same day as the odor tests. Prior to the blood tests, it was confirmed that patients did not have an active infection, as determined through clinical history and physical examination. Samples were collected during stable outpatient visits. The SII was calculated by multiplying the neutrophil count by the platelet count and dividing by the lymphocyte count, in accordance with established literature [35].

SII = Neutrophil × Platelet / Lymphocyte

**Table 1:** Demographic and clinical characteristics of atrial fibrillation and control groups

	**AF group (*n* ═ 43)**	**Control group (*n* ═ 42)**	***P***
Age (years)	50 [40–64]	47 [29–59]	0.128
Male gender, *n* (%)	15 (34.9)	16 (38.1)	0.750
Smoking, *n* (%)	18 (41.8)	17 (40.4)	0.870
Alcohol consumption, *n* (%)	9 (20.9)	6 (14.3)	0.571
Systolic blood pressure (mmHg)	135.9 ± 12.9	131.0 ± 12.0	0.073
Diastolic blood pressure (mmHg)	73.5 ± 9.7	72.1 ± 8.3	0.480
Average heart rate (beats/min)	80.8 ± 16.0	76.5 ± 16.2	0.230
Body mass index (kg/m^2^)	25.8 ± 2.3	26.3 ± 3.3	0.420
Hypertension, *n* (%)	36 (83.7)	28 (67.0)	0.083
Diabetes mellitus, *n* (%)	16 (37.2)	12 (29.0)	0.500
Hyperlipidemia, *n* (%)	11 (25.6)	7 (16.7)	0.430
Statin, *n* (%)	13 (30.2)	7 (16.7)	0.220
Anticoagulant/antiplatelet use, *n* (%)	41 (95.3)**^#^**	8 (19.0)**^†^**	<0.001^*^
β blocker use, *n* (%)	36 (83.7)	29 (69.0)	0.130
ACEI/ARB, *n* (%)	16 (37.2)	12 (29.0)	0.500

Data are presented as mean ± standard deviation or as number (percentage); non-normally distributed variables are reported as median with interquartile range. Continuous variables were compared using Student’s *t*-test (for normal distribution) or Mann–Whitney *U* test (for non-normal distribution). Categorical variables were analyzed using the chi-square test or Fisher’s exact test, as appropriate. *P < 0.05 was deemed statistically significant. ^#^Participants with a CHA_2_DS_2_-VA score (congestive heart failure, hypertension, age, diabetes mellitus, stroke, vascular disease) of 1 or more were prescribed anticoagulants. ^†^Furthermore, nineteen percent of the control group received only antiplatelet therapy. Abbreviations: ACE: Angiotensin converting enzyme inhibitors; AF: Atrial fibrillation; ARB: Angiotensin receptor blocker.

### Ethical statement

Ethical approval was obtained from the Trakya University Institutional Ethics Committee (decision number 2023/32). This study adhered to the Declaration of Helsinki, and written informed consent was secured from all participants.

### Statistical analysis

Statistical analyses were conducted using Statistical Package for the Social Sciences (SPSS) for Windows, version 26 (SPSS, Chicago, IL, USA). An a priori sample size calculation for the primary outcome, the TDI score, was performed using G*Power software (version 3.1.9.7), based on a two-sample independent means comparison (two-tailed *t*-test) with an allocation ratio of 1:1. This calculation was informed by a previous study reporting mean TDI scores in patients with heart failure and controls (16.4 ± 7.8 vs. 33.3 ± 5.2, *P <* 0.001) [23]. Anticipating that olfactory dysfunction in AF would be less pronounced than in heart failure, a conservative effect size (Cohen’s *d* ═ 0.80) was utilized. With a two-sided α of 0.05 and a statistical power of 95%, the minimum required sample size was determined to be 84 participants (42 per group). To accommodate potential exclusions or missing data, a total sample size of approximately 85–90 participants was targeted, resulting in 85 participants included in the final analysis (43 AF patients and 42 controls).

Normality was assessed using the Shapiro-Wilk test. Normally distributed continuous variables are presented as mean ± SD and compared using Student’s *t*-test; non-normally distributed variables are presented as median [IQR] and compared using the Mann–Whitney *U* test. Categorical variables were expressed as numbers and percentages, and the chi-square test or Fisher’s exact test was utilized for comparisons between groups. Effect sizes for key comparisons (TDI score and SII) were reported along with 95% confidence intervals, while the rank-biserial correlation was used as the effect size measure for Mann-Whitney *U* comparisons. All remaining analyses were labeled as exploratory/secondary to avoid overemphasis on multiple comparisons. No missing data were present for the analyzed variables, thus no imputation was necessary. Spearman tests were conducted to evaluate the correlation of odor subtests, TDI, and SII values with AF symptom severity (EHRA symptom scale). Correlation strength was interpreted based on the absolute value of the correlation coefficient: values between 0.1 and 0.3 were considered weak, between 0.3 and 0.5 moderate, and above 0.5 strong. A *P* value < 0.05 was accepted as indicative of statistical significance.

## Results

### Clinical and demographic characteristics of the study population

The demographic and clinical characteristics of the control and AF groups are summarized in Table 1. No statistically significant differences were observed between the groups concerning age, gender, arterial blood pressure, heart rate, or chronic diseases. However, the use of anticoagulant/antiplatelet medications was significantly higher in the AF group compared to the control group (41 [95.3%] vs. 8 [19.0%], *P <* 0.001). Within the AF group, patients with a CHA_2_DS_2_-VA score (congestive heart failure, hypertension, age, diabetes mellitus, stroke, vascular disease) of 1 or more were administered anticoagulants. In contrast, 19% of control group patients were taking antiplatelet agents, with none using anticoagulants; these patients were prescribed antiplatelets for non-obstructive coronary artery disease or, in the case of those with diabetes mellitus, for primary prevention of cardiovascular disease, as obstructive coronary artery disease was an exclusion criterion in our study. Laboratory, echocardiography, and olfactory test parameters are detailed in Table 2. No significant differences were observed between groups regarding fasting blood glucose, creatinine, liver function tests, thyroid-stimulating hormone, lipid profiles, left ventricular ejection fractions, or interventricular septum thickness. Neutrophil counts were significantly higher in the AF group compared to the control group (4.8 [2.4--13.2] vs. 4.0 [2.4--6], *P <* 0.001). Similarly, platelet counts were greater in the AF group (263 [142--421] vs. 180 [140–290], *P <* 0.001). Conversely, lymphocyte counts were lower in the AF group (1.4 ± 0.4 vs. 1.7 ± 0.5, *P ═* 0.003).

**Table 2:** Laboratory, echocardiographic, and olfactory test parameters of the study population

	**AF group (*n* ═ 43)**	**Control group (*n* ═ 42)**	***P***
Fasting blood glucose (mg/dL)	127.8 ± 45.7	119.7 ± 22.8	0.310
Creatinine (mg/dL)	1.10 ± 0.58	0.98 ± 0.39	0.270
Alanine aminotransferase, U/L	25.1 ± 3.6	24.2 ± 4.7	0.326
Aspartate aminotransferase, U/L	35.3 ± 5.6	36.3 ± 3.5	0.325
Thyroid stimulating hormone, mIU/mL	2.18 ± 0.44	2.26 ± 0.32	0.340
Sodium, mmol/L	137.5 ± 3.5	136.9 ± 2.8	0.385
Potassium, mmol/L	4.10 ± 0.80	4.00 ± 0.90	0.590
Triglyceride, mg/dL	110.9 ± 31.4	99.4 ± 33.7	0.110
Low-density lipoprotein, mg/dL	103.9 ± 34.2	98.4 ± 27.1	0.410
High-density lipoprotein, mg/dL	46.5 ± 12.6	47.1 ± 11.7	0.840
Hemoglobin (g/dL)	12.8 ± 1.7	13.2 ± 1.2	0.210
Platelet count (x10^3^/µL)	263 [142–241]	180 [140–290]	<0.001*
Neutrophil count (x10^3^/µL)	4.8 [2.4–13.2]	4.0 [2.4–6]	<0.001*
Lymphocyte count (x10^3^/µL)	1.4 ± 0.4	1.7 ± 0.5	0.003*
Systemic immune-inflammation index	878 [368–5769]	503 [243–1450]	0.007*
Left ventricular enddiastolic diameter, mm	50.8 ± 4.5	50.2 ± 4.0	0.520
Left ventricular ejection fraction, %	54.9 ± 3.2	55.3 ± 4.1	0.620
Interventricular septum thickness, mm	11.7 ± 1.5	11.3 ± 1.7	0.251
sPAP, mmHg	32.5 ± 5.4	31.6 ± 4.4	0.410
Odor threshold score	4 [1–8]	5 [2–10]	0.001*
Odor discrimination score	7 [4–12]	13 [8–16]	<0.001*
Odor identification score	7.7 ± 2.6	12.6 ± 1.7	<0.001*
TDI score	19 [10–29]	29.5 [25–39]	<0.001*

Data are presented as mean ± standard deviation for normally distributed variables and as median with interquartile range for non-normally distributed variables. Continuous variables were compared using Student’s *t*-test for normal distribution and Mann–Whitney *U* test for non-normal distribution. *A *P* value of < 0.05 was considered statistically significant. Abbreviations: AF: Atrial fibrillation; sPAP: Systolic pulmonary artery pressure; TDI: Threshold-Discrimination-Identification.

### Relationship between SII, odor tests and AF

In this study, the SII was significantly elevated in the AF group compared to the control group (878 [368–5769] vs. 503 [243–1450], *P ═* 0.007). The median Hodges-Lehmann difference between groups for SII was +265.7 (95% CI, +89.0 to +763.0; *P ═* 0.007), with a rank-biserial correlation of r ═ –0.340. The scores for the odor subtests—OT, OD, and OI—were significantly lower in the AF group compared to the control group (4 [1–8] vs. 5 [2–10], *P ═* 0.001; 7 [4–12] vs. 13 [8–16], *P <* 0.001; 7.7 ± 2.6 vs. 12.6 ± 1.7, *P <* 0.001, respectively). The TDI score was also significantly lower in AF patients than in the control group (19 [10–29] vs. 29.5 [25–39], *P <* 0.001). The Hodges-Lehmann estimator indicated a median difference of --13.0 between groups for the TDI score (95% confidence interval, --16.0 to --9.0), revealing a substantial group difference (rank-biserial correlation, r_rb_ ═ 0.664).

Correlation analysis identified strong negative correlations between the TDI score and the EHRA symptom scale (r ═ –0.748, *P <* 0.001) and SII (r ═ –0.412, *P ═* 0.006), as well as a positive correlation between the EHRA symptom scale and SII (*r* ═ 0.539, *P <* 0.001). Additionally, Sniffin’ Sticks subtests OT, OD, and OI scores demonstrated significant correlations with the EHRA symptom scale (Table 3).

**Table 3:** Correlation analysis results of odor tests, inflammation scores, and symptom severity in the AF group

	**EHRA**		**TDI**		**OT**		**OD**		**OI**	
	^**r**^	^**p**^	^**r**^	^**p**^	^**r**^	^**p**^	^**r**^	^**p**^	^**r**^	^**p**^
TDI	**--**0.748	<0.001*								
OT	**--**0.376	0.013*	0.568	<0.001*						
OD	**--**0.747	<0.001*	0.844	<0.001*	0.246	0.112				
OI	**--**0.772	<0.001*	0.814	<0.001*	0.256	0.098	0.572	<0.001*		
SII	0.539	<0.001*	**--**0.412	0.006*	**--**0.231	0.136	**--**0.268	0.082	**--**0.469	0.002*

Correlations were conducted exclusively within the AF group (*n* ═ 43). Given that the EHRA symptom scale is an ordinal variable, correlations involving the EHRA scale were assessed using Spearman’s rank correlation coefficient. *A *P* value of < 0.05 was deemed statistically significant. Abbreviations: AF: Atrial fibrillation; EHRA: European Heart Rhythm Association; OT: Odor threshold; OD: Odor discrimination; OI: Odor identification; SII: Systemic immune-inflammation index; TDI: Threshold-Discrimination-Identification.

## Discussion

The primary outcome of this study was the identification of olfactory dysfunction in patients with AF compared to a control group. Our findings revealed significant reductions in all olfactory subtests and the total score on the TDI test among AF patients. Prior to this study, there had been no direct examinations of olfactory dysfunction within this patient population; existing knowledge was derived from studies investigating different hypotheses. A recent article indicated that olfactory dysfunction notably increased the risk of stroke following approximately ten years of follow-up in patients who underwent baseline olfactory testing [36]. Sub-analysis of this study highlighted that pronounced olfactory dysfunction was prevalent among patients with comorbidities such as heart failure, coronary artery disease, and AF. Additionally, the baseline olfactory test utilized was the 12-item Sniffin’ Sticks, which is favored for community screening due to its simplicity. In contrast, our study directly assessed olfactory function in AF patients using an extended version of the Sniffin’ Sticks test for evaluation.

Emerging evidence suggests that comorbidities, particularly heart failure, are frequently observed in asymptomatic AF patients, notably those with a convex-shaped chest wall and increased anteroposterior thoracic diameter [37]. A retrospective investigation into olfactory anatomical abnormalities and cardiac arrhythmias demonstrated significantly lower olfactory bulb volume and olfactory sulcus depth on cranial MRI in both the cardiac arrhythmia and COVID-19 groups compared to controls [38]. Within this study, AF was the most prevalent arrhythmia disorder. Furthermore, our study did not employ any quantitative diagnostic tests to evaluate olfactory function in AF patients. Notably, during the COVID-19 pandemic, cardiac involvement was reported to be nine times higher than in typical viral infections, with a parallel decline in olfactory function observed across subsequent waves of the pandemic [39, 40]. Interestingly, a study conducted prior to the pandemic found an association between olfactory dysfunction and heart failure, correlating the severity of olfactory dysfunction with heart failure severity [23]. Similar to our research, this study directly examined the relationship between heart failure and olfactory dysfunction, utilizing Sniffin’ Sticks as a quantitative measure. In our study, the EHRA scale, which assesses symptom severity in AF patients, was correlated with the degree of olfactory dysfunction. A study published last year demonstrated that poor olfactory function conferred an increased risk for heart failure over approximately eight years of follow-up [41]. In a subsequent publication by the same researchers, a link was established between poor olfactory performance and newly diagnosed heart failure, underscoring olfactory dysfunction as a potential novel marker for heart failure diagnosis [25].

The development of new diagnostic methods for AF, a condition that can be paroxysmal, asymptomatic, and capable of causing silent strokes, is critical due to its significant mortality rate [5–9]. Research has indicated that olfactory dysfunction correlates directly with mortality and serves as one of the strongest predictors of five-year mortality, particularly in older populations [42, 43]. Moreover, in neurodegenerative diseases such as Parkinson’s, olfactory dysfunction emerges as a prodromal symptom preceding motor deficits [44]. Since our study was cross-sectional, we could not ascertain whether olfactory dysfunction serves as a prodromal symptom in AF patients. A recent study evaluating cardiac sympathetic denervation in Parkinson’s disease patients using 123I-meta-iodobenzylguanidine myocardial scintigraphy reported an association between olfactory dysfunction and cardiac sympathetic burden [45].

Microembolism, cerebral hypoperfusion, inflammatory processes, and elevated proinflammatory cytokine levels are mechanisms likely contributing to the development of olfactory dysfunction in AF patients. It has been suggested that similar mechanisms may underlie olfactory dysfunction in heart failure, and olfactory tests could serve as diagnostic tools in cardiocerebral syndrome [23, 41, 46]. The role of proinflammatory cytokines, particularly interleukin-6 (IL-6), in the pathophysiology of both AF and olfactory dysfunction is well-documented [47, 48]. In our study, we employed the SII, known to correlate positively with IL-6 levels in autoimmune diseases, to investigate the role of inflammation in olfactory dysfunction among AF patients [49]. Our findings indicated that SII was elevated in the AF group, demonstrating a positive correlation with AF symptom severity, while showing a negative correlation with odor test scores. SII serves as an indirect marker of systemic inflammation, suggesting an association between higher SII, AF, and poor olfactory function, though not establishing a causal or pathophysiological relationship. The presence of cognitive dysfunction in patients with both AF and olfactory dysfunction, alongside observed deterioration in white matter integrity on cranial MRI, implies that vascular events may contribute to olfactory dysfunction in AF patients [18, 19, 50, 51].

Our study has several limitations. Although patients with cerebrovascular disease were excluded, cranial MRI could not be conducted to elucidate potential mechanisms, particularly silent microembolisms, that may explain olfactory dysfunction in AF patients. Given that silent AF is rare in individuals under 65, current guidelines do not advocate for ECG Holter monitoring in asymptomatic individuals under this age. While our control group consisted of asymptomatic participants below 65, future research could benefit from employing ECG Holter monitors for screening purposes. Due to the time constraints and costs associated with olfactory testing, as well as exclusion criteria, particularly regarding participant age, we included patients with paroxysmal AF who exhibited AF on their ECG during outpatient visits. Consequently, this study may not fully represent the relationship between AF and olfactory dysfunction in older adults. Future studies investigating AF and olfactory dysfunction in patients aged 65 and older will enhance our understanding of this relationship. The optimal approach to test this hypothesis would involve comparing newly diagnosed AF patients with a control group and conducting longitudinal follow-up. However, we included both newly diagnosed and previously diagnosed AF patients in our study. The higher utilization of anticoagulants in the AF group compared to the control group may have influenced the SII results. Additionally, SII was calculated from a single complete blood count sample, limiting our ability to assess changes over time. We were also unable to measure C-reactive protein or sedimentation rates to determine the presence of acute infection or inflammation during SII evaluation. Patients in the AF group received anticoagulant therapy, while those in the control group were treated with antiplatelet therapy. Ideally, future studies should select a control group comprising individuals not on any antiplatelet therapy. Including only newly diagnosed AF patients in multi-center studies could further clarify this topic. Furthermore, although previous studies have shown a correlation between IL-6 and SII values, IL-6 levels were not evaluated in our study.

## Conclusion

In this study, olfactory function was significantly reduced in patients with AF compared to the control group. Smell test scores were negatively correlated with symptom severity in AF patients. Additionally, SII was found to be associated with both AF and olfactory dysfunction. This single-center case-control study demonstrates that olfactory dysfunction and elevated SII are related to AF and symptom burden. Larger prospective studies with standardized rhythm monitoring are necessary to assess potential screening or diagnostic utility.

## Data Availability

The data collected in this study, along with the accompanying analyses, are presented in the article. Additionally, the data can be obtained from the authors upon request.

## References

[1] Linz D, Gawalko M, Betz K, Hendriks JM, Lip GYH, Vinter N (2024). Atrial fibrillation: epidemiology, screening and digital health. Lancet Reg Health Eur.

[2] Van Gelder IC, Rienstra M, Bunting KV, Casado-Arroyo R, Caso V, Crijns HJ (2024). 2024 ESC Guidelines for the management of atrial fibrillation developed in collaboration with the European Association for Cardio-Thoracic Surgery (EACTS). Eur Heart J.

[3] Mensah GA, Fuster V, Murray CJL, Roth GA (2023). Global burden of cardiovascular diseases and risks collaborators. Global burden of cardiovascular diseases and risks, 1990–2022. J Am Coll Cardiol.

[4] Babela R, Baráková A, Hatala R (2024). Epidemiology and comprehensive economic impact of atrial fibrillation and associated stroke in Slovakia. BMC Health Serv Res.

[5] Peng X, Li Q, Liu X, He L, Liu N, Yuan C (2025). Asymptomatic atrial fibrillation: clinical characteristics, outcomes, and prognostic impact of rhythm control. Heart Rhythm.

[6] Harwood S, Shoemaker MB, Barnard J, Van Wagoner DR, Morin DP, Chung MK (2025). Genomics in atrial fibrillation: predicting recurrence of atrial fibrillation after treatment using genetics. Prog Cardiovasc Dis.

[7] Saksena S, Ken-Opurum J, McKindley DS, Preblick R, Rashkin J, Aldaas OM (2025). Arrhythmia recurrence and rhythm control strategies after catheter ablation of newly diagnosed atrial fibrillation (ARRC-AF Study). JACC Clin Electrophysiol.

[8] Shi Y, Zhang Z, Zhang T, Zhang L, An S, Chen Y (2024). Circulating soluble suppression of tumorigenicity-2 and the recurrence of atrial fibrillation after catheter ablation: a meta-analysis. Biomol Biomed.

[9] Lee WC, Lin YW, Shih JY, Chen ZC, Wu NC, Chang WT (2025). Dapagliflozin and Sirtuin-1 interaction and mechanism for ameliorating atrial fibrillation in a streptozotocin-induced rodent diabetic model. Biomol Biomed.

[10] Fekete M, Liotta EM, Molnar T, Fülöp GA, Lehoczki A (2025). The role of atrial fibrillation in vascular cognitive impairment and dementia: epidemiology, pathophysiology, and preventive strategies. Geroscience.

[11] Zhang S, Zhang N, Liu L, Zheng W, Ma ZL, Qiao SY (2024). Global epidemiology of mental disorder in atrial fibrillation between 1998–2021:a systematic review and meta-analysis. World J Psychiatry.

[12] Rivard L, Friberg L, Conen D, Healey JS, Berge T, Boriani G (2022). Atrial fibrillation and dementia: a report from the AF-SCREEN International Collaboration. Circulation.

[13] Koh YH, Lew LZW, Franke KB, Elliott AD, Lau DH, Thiyagarajah A (2022). Predictive role of atrial fibrillation in cognitive decline: a systematic review and meta-analysis of 2.8 million individuals. Europace.

[14] Hong CT, Chan L, Wu D, Chen WT, Chien LN (2019). Association between Parkinson’s disease and atrial fibrillation: a population-based study. Front Neurol.

[15] Dan X, Wechter N, Gray S, Mohanty JG, Croteau DL, Bohr VA (2021). Olfactory dysfunction in aging and neurodegenerative diseases. Ageing Res Rev.

[16] Nattel S, Heijman J, Zhou L, Dobrev D (2020). Molecular basis of atrial fibrillation pathophysiology and therapy: a translational perspective. Circ Res.

[17] Xie Y, Wang S, Cha X, Li F, Xu Z, Wu J (2025). Aging and chronic inflammation: impacts on olfactory dysfunction—a comprehensive review. Cell Mol Life Sci.

[18] Fu X, Xie B, Li L, Hao Y, Yang S, Guo C (2025). Olfactory identification impairment in cerebral small-vessel disease indicates cognitive network disconnection and predicts accelerated cognitive decline. J Neurol.

[19] Petersen M, Chevalier C, Naegele FL, Ingwersen T, Omidvarnia A, Hoffstaedter F (2024). Mapping the interplay of atrial fibrillation, brain structure, and cognitive dysfunction. Alzheimers Dement.

[20] Chen YC, Liu CC, Hsu HC, Hung KC, Chang YJ, Ho CN (2024). Systemic immune-inflammation index for predicting postoperative atrial fibrillation following cardiac surgery: a meta-analysis. Front Cardiovasc Med.

[21] Liu F, Ran Q, Zhang H, Chen J (2025). The systemic immune-inflammation index and the risk of Parkinson’s disease in the U.S.: a cross-sectional study. J Clin Med.

[22] Liang L, Su Q (2025). Systemic immune-inflammation index and the short-term mortality of patients with sepsis: a meta-analysis. Biomol Biomed.

[23] Akşit E, Çil ÖÇ (2020). Olfactory dysfunction in patients with ischemic heart failure. Acta Cardiol Sin.

[24] Chamberlin KW, Li C, Kucharska-Newton A, Luo Z, Reeves M, Shrestha S (2025). Olfaction and coronary heart disease. JAMA Otolaryngol Head Neck Surg.

[25] Chamberlin KW, Yuan Y, Li C, Luo Z, Reeves M, Kucharska-Newton A (2024). Olfactory impairment and the risk of major adverse cardiovascular outcomes in older adults. J Am Heart Assoc.

[26] Leon M, Troscianko ET, Woo CC (2024). Inflammation and olfactory loss are associated with at least 139 medical conditions. Frontiers in Molecular Neuroscience.

[27] Gore MR (2020). Association of olfactory neuropathy spectrum disorder and Wolff-Parkinson-White syndrome: a report of a case. Clin Case Rep.

[28] Maruyama T, Yasuda S, Odashiro K, Kaji Y, Harada M (2007). Anosmia induced by amiodarone. Am J Med.

[29] Che X, Li Y, Fang Y, Reis C, Wang H (2018). Antiarrhythmic drug-induced smell and taste disturbances: a case report and literature review. Medicine (Baltimore).

[30] Khanna D, Peltzer C, Kahar P, Parmar MS (2022). Body Mass Index (BMI): a screening tool analysis. Cureus.

[31] Camm AJ, Kirchhof P, Lip GY, Schotten U, Savelieva I, Ernst S (2010). Guidelines for the management of atrial fibrillation: the Task Force for the Management of Atrial Fibrillation of the European Society of Cardiology (ESC). Europace.

[32] Oleszkiewicz A, Schriever VA, Croy I, Hähner A, Hummel T (2019). Updated Sniffin’ Sticks normative data based on an extended sample of 9139 subjects. Eur Arch Otorhinolaryngol.

[33] Haehner A, Mayer AM, Landis BN, Pournaras I, Lill K, Gudziol V (2009). High test-retest reliability of the extended version of the “Sniffin’ Sticks” test. Chem Senses.

[34] Hedner M, Larsson M, Arnold N, Zucco GM, Hummel T (2010). Cognitive factors in odor detection, odor discrimination, and odor identification tasks. J Clin Exp Neuropsychol.

[35] Hu B, Yang XR, Xu Y, Sun YF, Sun C, Guo W (2014). Systemic immune-inflammation index predicts prognosis of patients after curative resection for hepatocellular carcinoma. Clin Cancer Res.

[36] Chamberlin KW, Li C, Kucharska-Newton A, Luo Z, Reeves M, Shrestha S (2025). Poor olfaction and risk of stroke in older adults: the Atherosclerosis Risk in Communities Study. Stroke.

[37] Sonaglioni A, Grasso E, Nicolosi GL, Lombardo M (2024). Modified Haller Index is inversely associated with asymptomatic status in atrial fibrillation patients undergoing electrical cardioversion: a preliminary observation. Minerva Cardiol Angiol.

[38] Gore MR (2022). Olfactory radioanatomical findings in patients with cardiac arrhythmias, COVID-19, and healthy controls. Cureus.

[39] Akşit E, Köder A (2023). Is there a relationship between olfactory dysfunction and decreased thromboembolic events after the first wave of the COVID-19 pandemic?. Balkan Med J.

[40] Akşit E, Çil ÖÇ, Kaya H (2020). Olfactory dysfunction may predict myocardial injury in COVID-19 patients. Med Hypotheses.

[41] Chamberlin KW, Li C, Kucharska-Newton A, Luo Z, Reeves M, Shrestha S (2025). Poor olfaction and risk of heart failure in the Atherosclerosis Risk in Communities Study. J Gerontol A Biol Sci Med Sci.

[42] Ruane R, Lampert O, Larsson M, Vetrano DL, Laukka EJ, Ekström I (2025). Olfactory deficits and mortality in older adults. JAMA Otolaryngol Head Neck Surg.

[43] Pinto JM, Wroblewski KE, Kern DW, Schumm LP, McClintock MK (2014). Olfactory dysfunction predicts 5-year mortality in older adults. PLoS One.

[44] Bang Y, Lim J, Choi HJ (2021). Recent advances in the pathology of prodromal non-motor symptoms olfactory deficit and depression in Parkinson’s disease: clues to early diagnosis and effective treatment. Arch Pharm Res.

[45] Ryu DW, Yoo SW, Choi KE, Oh YS, Kim JS (2024). Correlation of olfactory function factors with cardiac sympathetic denervation in Parkinson’s disease. J Neurol.

[46] Akşit E, Yıldırım ÖT (2020). Which quantitative test can predict cardiocerebral syndrome in patients with heart failure?. Int J Cardiol.

[47] Yu JF, Dong Q, Du YM (2025). Interleukin-6:molecular mechanisms and therapeutic perspectives in atrial fibrillation. Curr Med Sci.

[48] Song XY, Mou YK, Wang HR, Wang Y, Liu WC, Yang T (2025). Interleukin-6 and olfactory dysfunction: focus on changes, effects, and mechanisms. Mediators Inflamm.

[49] Zeb S, Khan Z, Ashraf Javaid M, Rumman Swati MAA (2024). Relationship between serum interleukin-6 levels, systemic immune-inflammation index, and other biomarkers across different rheumatoid arthritis severity levels. Cureus.

[50] Berman JP, Norby FL, Mosley T, Soliman EZ, Gottesman RF, Lutsey PL (2019). Atrial fibrillation and brain magnetic resonance imaging abnormalities. Stroke.

[51] Bothwell AR, Resnick SM, Ferrucci L, Tian Q (2023). Associations of olfactory function with brain structural and functional outcomes: a systematic review. Ageing Res Rev.

